# High OX-40 expression in the tumor immune infiltrate is a favorable prognostic factor of overall survival in non-small cell lung cancer

**DOI:** 10.1186/s40425-019-0827-2

**Published:** 2019-12-16

**Authors:** Erminia Massarelli, Vincent K. Lam, Edwin R. Parra, Jaime Rodriguez-Canales, Carmen Behrens, Lixia Diao, Jing Wang, Jorge Blando, Lauren A. Byers, Niranjan Yanamandra, Sara Brett, Peter Morley, Padmanee Sharma, James Allison, Ignacio I. Wistuba, John V. Heymach

**Affiliations:** 1 Department of Thoracic Head and Neck Medical Oncology, The University of Texas MD Anderson Cancer Center, Houston, TX USA; 2 Department of Medical Oncology, City of Hope Comprehensive Cancer Center, Duarte, CA USA; 3 Department of Translational Molecular Pathology, The University of Texas MD Anderson Cancer Center, Houston, TX USA; 4 Department of Bioinformatics and Computational Biology, The University of Texas MD Anderson Cancer Center, Houston, TX USA; 5 Department of Immunology, The University of Texas MD Anderson Cancer Center, Houston, TX USA; 6 Immuno-Oncology and Combinations DPU, GlaxoSmithKline, Collegeville, PA USA; 7 Biopharm Molecular Discovery Medicines Research Centre, Gunnels Wood Road, Stevenage, Hertfordshire, SG1 2NY UK; 8 Sidney Kimmel Comprehensive Cancer Center, Sidney Kimmel Comprehensive Cancer Center, Johns Hopkins University, Baltimore, MD USA

**Keywords:** OX-40, Lung cancer, NSCLC, Immune checkpoint; immunotherapy

## Abstract

**Introduction:**

OX-40 co-stimulatory signaling plays a role in mounting anti-tumor immune responses and clinical trials targeting this pathway are ongoing. However, the association of with OX-40 protein expression with clinical outcomes and pathological features in non-small cell lung cancer (NSCLC) are largely unknown.

**Methods:**

Surgically-resected stage I-III NSCLC specimens (*N* = 100) were stained by immunohistochemistry (IHC) for the following immune markers: OX-40, PD-L1, PD-1, CD3, CD4, CD8, CD45RO, CD57, CD68, FOXP3, granzyme B, and ICOS. Immune-related markers mRNA expression were also assessed. We evaluated the association of OX-40 levels with major clinicopathologic variables, including molecular driver mutations.

**Results:**

OX-40 IHC expression was observed in all tested tumors, predominantly localized in the membrane of the tumor immune infiltrate, and was not associated with a specific clinicopathologic or molecular subtype. High OX-40 expression levels measured by IHC median score were associated with better overall survival (OS) (*p* = 0.002), independent of CD3/CD8, PD-L1, and ICOS expression. High OX-40 IHC score was associated with increased expression of immune-related genes such as CD3, IFN-gamma, ICOS, CD8, CXCL9, CXCL10, CCL5, granzyme K.

**Conclusions:**

High OX-40 IHC expression in the tumor immune infiltrate is associated with favorable prognosis and increased levels of immune-related genes including IFN-gamma in patients with surgically resected stage I-III NSCLC. Its prognostic utility is independent of PD-L1 and other common markers of immune activation. High OX-40 expression potentially identifies a unique subgroup of NSCLC that may benefit from co-stimulation with OX-40 agonist antibodies and potentially enhance the efficacy of existing immune checkpoint therapies.

## Introduction

Over the last decade, encouraging progress has been made in the treatment of advanced/metastatic non-small cell lung cancer (NSCLC) patients. Immune checkpoint blockade via PD(L)-1 inhibition is currently approved by the Food and Drug Administration (FDA) as second-line treatment for metastatic NSCLC based on the overall survival (OS) benefit compared to standard of care chemotherapy [1–3]. More recently, pembrolizumab was approved as frontline treatment for metastatic NSCLC PDL-1 positive patients on the basis of a significant improvement when compared to standard platinum-based chemotherapy, both in response rate (45% versus 28%) and overall survival (10.3 months versus 6 months) [4]. However, the majority of patients with advanced NSCLC still do not benefit from immune checkpoint inhibition. In addition to targeting immune inhibitory receptors such as PD-1, generating optimal anti-tumor response also requires T-cell receptor activation plus co-stimulation, such as by tumor necrosis factor receptor family members (TNFRSF), OX-40 (CD134), and 4-1BB (CD137) [5, 6]. OX-40 (TNFRSF4/CD134) is a 50-kDa type-I membrane glycoprotein expressed on activated CD4+ and CD8+ T cells and has been shown to be the sole receptor for the OX-40 ligand [7]. The interaction between OX-40 and the OX-40-ligand supplies a co-stimulatory signal for T-cell proliferation in a CD28-independent manner [8] in autoimmune diseases [9] and graft-versus-host disease [10]. It is of particular interest as treatment with an activating (agonist) anti-OX-40 monoclonal antibody (mAb) augments T-cell differentiation and cytolytic function leads to enhanced anti-tumor immunity against a variety of tumors [11]. OX-40 expression on tumor-infiltrating lymphocytes (TIL) correlates with improved survival in several human cancers such as cutaneous melanoma and colorectal cancer, suggesting that OX-40 signals may play a critical role in establishing an anti-tumor immune response [12, 13].

Ample pre-clinical studies have shown that targeting the OX-40 receptor suppresses tumor growth by increasing effector T-cell differentiation and proliferation, and by diminishing regulatory T-cell activity [14–17]. Multiple OX-40 agonists are currently under clinical investigation. Results from the first phase I trial of a murine IgG1 anti-OX-40 monoclonal antibody showed potent immune activation but with limited anti-tumor activity [18]. Thus, strategies exploring complementary approaches are of great interest, including the combination of anti-OX-40 with radiation or immune checkpoint inhibitors [11, 19]. The sequence and timing of these combinations may be important, as some pre-clinical models have suggested that concurrent use of PD-1 blockade can abrogate anti-OX-40 efficacy [20, 21].

Ongoing research efforts are aimed at unveiling predictive biomarkers of sensitivity to immunotherapy. Diverse studies indicate that in tumors, immune cell subpopulations are strategically distributed within different tissue compartments [22]. Consistent with findings in tumors from different locations and tissue types, increased total TILs have been associated with longer survival in both early-stage and advanced NSCLC [23–25]. However, studies measuring single-cell subtypes using immunohistochemistry (IHC) have reported conflicting results with one showing an association between increased CD8+ cytotoxic T cells (but not of CD4+ cells) and longer survival [26] and others showing the opposite results [27, 28]. In addition, Hiraoka et al. reported an absence of survival benefit of either elevated CD8+ or CD4+ TILs alone, but a statistically significant (and independent) prognostic effect of combined high stromal CD8+ and CD4+ in 109 NSCLC samples [29]. More recently, Schalper et al. have provided evidence that elevated CD3+ and CD8+ T cells is consistently associated with improved survival, but only CD8 provides independent prognostic information in NSCLC [30]. Therefore, objective measurement of TIL subpopulations could be useful to predict response or evaluate the local immune effect of anti-cancer immune drugs.

The goal of our study was to determine the clinical and pathological features of patients with surgically resected stage I-III NSCLC based on OX-40 expression and to explore the correlations of OX-40 expression by IHC and mRNA levels with other markers of immune activation/suppression. In addition, we explored the prognostic significance of co-expression of OX-40/PD-L1 and OX-40/ICOS in the immune infiltrate in a subgroup of NSCLC samples, based on prior evidence of the potential role of these two T-cell markers as predictive markers of response to checkpoint inhibitor therapy in solid tumors [31–35].

## Material and methods

### Tissue specimens

One hundred formalin-fixed paraffin-embedded (FFPE) specimens from surgically resected NSCLC (61 adenocarcinoma and 39 squamous cell carcinoma histology) were selected among the NSCLC patients included in the Profiling of Resistance patterns and Oncogenic Signaling Pathways in Evaluation of Cancers of the Thorax (PROSPECT) cohort. Clinical characteristics of these 100 patients are summarized in Table 1.

**Table 1:** Clinicopathologic and molecular characteristics

Characteristic	Number (*n* = 100)
Median age (range)	66 (41–84)
Sex	
Male	52
Female	48
Race	
Caucasian	90
Other	10
Smoking	
Never	7
Ever	93
Stage	
I	48
II	27
III	25
Histology	
Adenocarcinoma	61
Squamous	39
*EGFR*	
Mutated	7
Wild-type	45
*KRAS*	
Mutated	39
Wild-type	21
Not available	40
Recurrence	45

From all analyzed cases, FFPE tissue specimens were selected from the pathology files at MD Anderson Cancer Center. From each tissue block, a hematoxylin & eosin (H&E) stained slide was examined by a thoracic pathologist to evaluate the presence of tumor. Four microns-thick sections were cut from a representative tumor block selected from each case for immunohistochemistry (IHC) analysis. *EGFR* and *KRAS* mutation data obtained using Sanger sequencing were available in 94 cases. This study was approved by the MD Anderson Institutional Review Board.

### Immunohistochemistry

IHC was performed using an automated staining system (Bond Max, Leica Biosystems, Vista, CA, USA) with primary antibodies against OX-40 (activated T cells; mouse monoclonal, clone ACT-35, dilution 1:100, eBioscience, San Diego, CA, USA), PD-L1 (rabbit monoclonal, clone E1L3N, dilution 1:100, Cell Signaling, Technology, Beverly, MA, USA), PD-1 (rabbit monoclonal, clone EPR4877, dilution 1:250, Abcam, Cambridge, MA, USA), CD3 (T cell lymphocytes; rabbit polyclonal, dilution 1:100, DAKO, Carpinteria, CA, USA), CD4 (helper T cell; mouse monoclonal, clone 4B12, dilution 1:80, Leica Biosystems, Buffalo Grove, IL, USA), CD8 (cytotoxic T cell; mouse monoclonal, clone C8/144B, dilution 1:20, Thermo Fisher, Waltham, CA, USA), CD45RO (memory T cell; mouse monoclonal, clone UCHL1, ready to use; Leica Biosystems), CD57 (natural killer T cell; mouse monoclonal, clone HNK-1, dilution 1:40; BD Biosciences, San Jose, CA), CD68 (macrophages; mouse monoclonal, clone PG-M1, dilution 1:450, DAKO), FOXP3 (regulatory T cell; mouse monoclonal, clone 206D, dilution 1:50; Biolegend, San Diego, CA, USA), granzyme B (cytotoxic lymphocytes; mouse monoclonal, clone 11F1, ready to use, Leica Biosystems), and ICOS (activated T cells; rabbit monoclonal, dilution 1:100, Spring Bioscience). All slides were stained using previously optimized conditions including positive and negative controls (human embryonic kidney 293 cell line transfected and non-transfected with PD-L1 gene, and human placenta for PD-L1; human tonsil for the rest of the markers) and a non-primary antibody for negative control. Expression of all the markers in cells was detected using a Novocastra Bond Polymer Refine Detection kit (Leica Biosystems), with a diaminobenzidine (DAB) reaction to detect antibody labeling and hematoxylin counterstaining.

### Scanning and digital image analysis of immune markers

All the IHC stained slides were digitally scanned at 200x magnification into a high-resolution digital image of the whole tissue (e-slide manager) using a pathology scanner (Aperio AT Turbo, Leica Biosystems, Buffalo Grove, IL). The images were visualized using the ImageScope software program (Leica Biosystems) and analyzed using the Aperio Image Toolbox and GENIE analysis tool (Leica Biosystems). The densities of immune cells markers including PD-1, ICOS, OX-40 CD3, CD4, CD8, CD57, granzyme B, CD45RO, and FOXP3 were evaluated using the Aperio nuclear algorithm, CD68 using Aperio cytoplasmic algorithm, and counting the cells positive for them in five square areas (1 mm^2^ each) in the inside of the tumor compartment. Each area examined was overlapped with the sequential IHC slides to quantify each marker at the same location of the tumor specimen [36]. The average of total number of cells positive for each marker in the five square areas was expressed in density per mm^2^.

### PROSPECT gene analysis

The Illumina beadarray data were processed using the Model-Based Background Correction (MBCB) method (Xie, Bioinformatics; Ding, NAR) and quantile-quantile normalization as reported elsewhere [37–41]. All gene expression values were log2 transformed. The gene expression data has been archived at the Gene Expression Omnibus repository (GSE42127).

### Statistical analysis

Spearman correlation was used to determine the correlation between continuous variables of gene expression levels and OX-40 IHC levels. The top 100 probe sets were selected to create a heatmap. Spearman correlation test was used to determine the association between OX-40 IHC density and immune-related gene expression levels. Log-rank test was used to determine the association between different groups and survival. In the multivariate analysis, we included OX-40 density, gender, age, smoking pack-years, stage, histology, and adjuvant therapy in the Cox model to test the association between different groups and survival.

## Results

### OX-40 protein expression

**Fig. 1:**
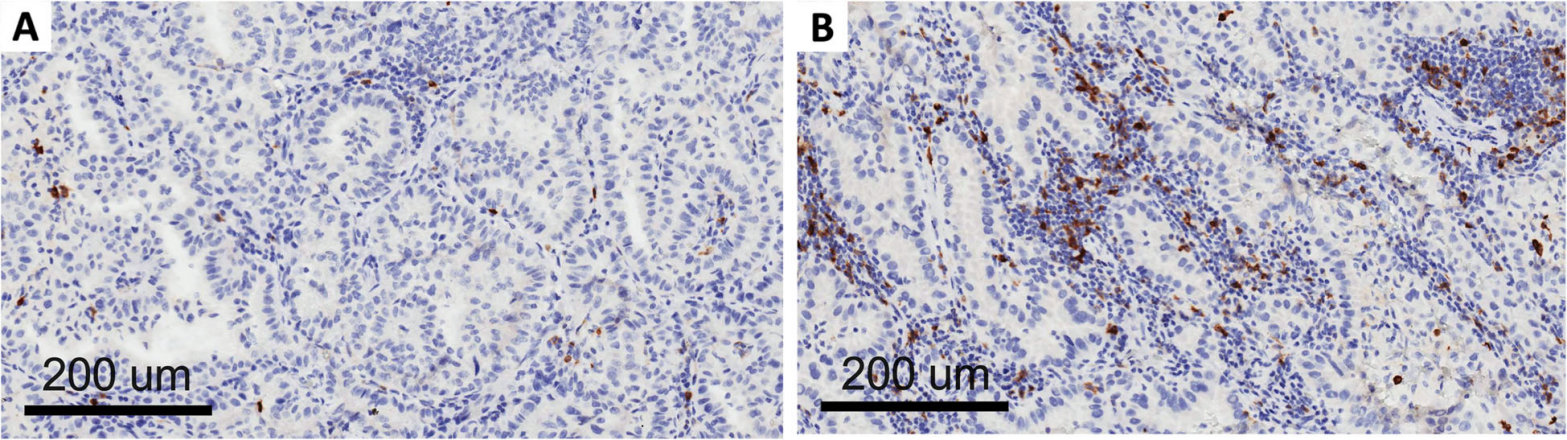
OX-40 expression on tumor infiltrating lymphocytes: low (**a**) and high expression (**b**)

Clinico-pathological and molecular data on the patients included in this study are shown in Table 1. OX-40 protein expression was localized in the membrane of the tumor immune infiltrating cells in the NSCLC samples (Fig. 1). The density score ranged from 56 to 1246 with a median value of 271 (standard deviation = 245). When the median value was used as cut-off of positivity, there was no statistical correlation between OX-40 IHC expression and clinico-pathological characteristics such as sex, smoking status, stage, and histology (data not shown). There was also no correlation between OX-40 protein expression and *EGFR* or *KRAS* mutation status in our study. OX-40 levels positively correlated with markers of immune activation and proliferation tested by IHC (Additional file 1: Table S1). A strong correlation between OX-40 and FOXP3 IHC was also observed (rho = 0.691, *p* < 0.0001). These findings are consistent with knowledge that OX-40 can be expressed in both activated T effector cells and T regulatory cells.

### Correlation between OX-40 protein expression and NSCLC prognosis

**Fig. 2:**
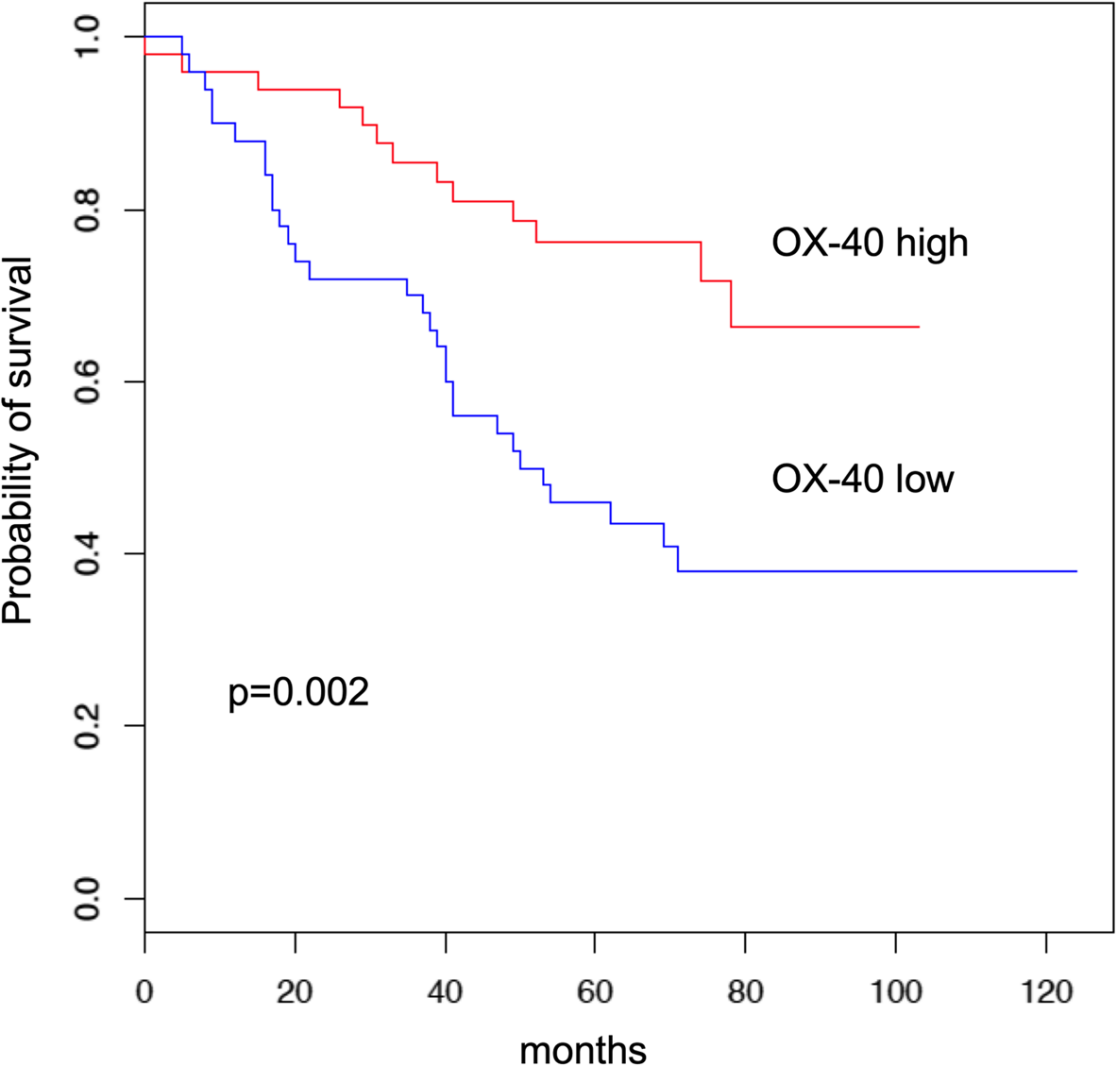
Overall survival Kaplan-Meier curves by OX-40 protein expression median value

Patients whose tumor samples showed higher OX-40 expression levels by density median score in the immune cells had a longer overall survival (OS) compared to those with low OX-40 expression (HR = 2.68 [95% CI 1.4–5.2], *p* = 0.002; Fig. 2a). This favorable prognostic effect was seen in both adenocarcinoma and squamous cell carcinoma, though it did not reach statistical significance in the adenocarcinoma subgroup (*p* = 0.08 and *p* = 0.04, respectively) (Additional file 1: Figure S1). In the multivariate model, OX-40 expression retained its prognostic role (*p* = 0.004) along with stage, histology, and adjuvant therapy.

To understand the prognostic significance of co-expression of OX-40 and other known immune-related prognostic IHC markers, we conducted univariate Cox regressions within OX-40-high subgroup. CD3/CD8 (*p* = 0.671), PD-L1 (*p* = 0.697), and ICOS (*p* = 0.491) were not associated with overall survival in this subgroup, suggesting that OX-40 has independent prognostic value. This is visualized by the Kaplan-Meier plots of OX-40 co-expression with these other immune IHC markers (Additional file 1: Figure S2).

### Correlation between OX-40 protein and immune-related genes

**Fig. 3:**
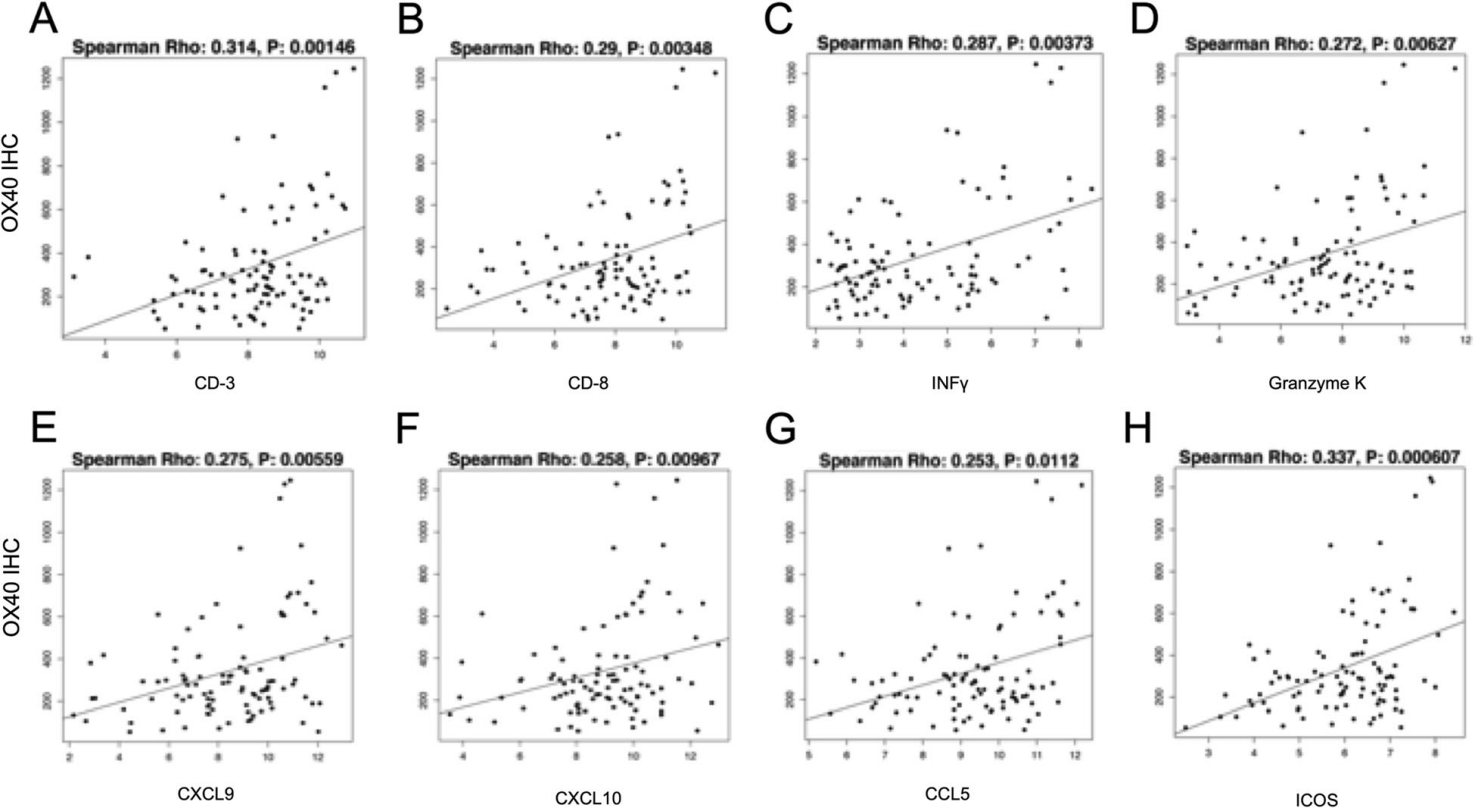
Correlation between OX-40 protein expression and gene expression levels of multiple markers of immune inflammation: CD-3 (**a**), CD-8 (**b**), IFN-gamma (**c**), granzyme K (**d**), CXCL9 (**e**), CXCL10 (**f**), CCL5 (**g**), and ICOS (**h**)

To characterize the activated pathways in OX-40-positive tumor samples, we conducted an analysis of mRNA expression stratified by OX-40 IHC expression. This was supported by the fact that OX-40 IHC protein expression correlated with OX-40 gene expression (*p* = 0.002). When analyzing the correlation between OX-40 IHC levels and mRNA expression of immune-related genes, we found the following markers of immune inflammation to have a highly significant positive association (*p* ≤ 0.01): CD3, CD8, IFN-gamma, ICOS, CXCL9, CXCL10, CCL5, and granzyme K (Fig. 3).

## Discussion

OX-40 is a co-stimulatory member of the tumor necrosis factor receptor superfamily expressed on activated CD4+ and CD8 + T cells [7]. In this study, we identified that high OX-40 protein expression by IHC in immune cell infiltrate of tumor samples from patients with surgically resected stage I-IIIA NSCLC has prognostic significance for improved OS. The association of OX-40 with prognosis has varied across different types of cancers. There is already evidence in the literature that OX-40 expression on TILs correlates with better survival in human cancers including malignant melanoma and colorectal cancer [12, 42]. On the other hand, OX-40 expression in other cancers such as cutaneous squamous cell carcinoma and hepatocellular carcinoma is associated with a poorer prognosis and an immunosuppressive tumor microenvironment [43, 44]. Our study is the first to report in the literature on OX-40 as a prognostic marker for favorable outcome in NSCLC.

The presence of CD3+ and CD8+ tumor infiltrating cells have previously been shown to be associated with survival in NSCLC [30]. In our study, we have shown that the cohort of NSCLC patients whose tumor samples express high OX-40 IHC density staining in the immune cell infiltrate have a survival advantage independent of CD3+/CD8+ expression. We observed the same independent prognostic characteristic of OX-40 when we evaluated the impact of PD-L1 co-expression. This suggests that OX-40 is a stronger driver of prognosis than PD-L1 in early stage NSCLC. This finding is of particular interest because of the ongoing clinical development of OX-40 agonists, alone or in combination with PD-1/PD-L1 inhibitors, in the treatment of solid tumors including advanced NSCLC. The rationale for this combination is also supported by recent evidence that OX-40 agonist monotherapy can induce PD-L1 expression in the tumor immune infiltrate and tumoral cells [35]. An important issue that remains unanswered is whether these subgroups defined by OX-40 and PD-L1 will have different degrees of benefit to treatment with OX-40 and PD-L1 inhibitors.

Another important marker of T cell activation is CD278 or ICOS (inducible T-cell costimulator), a member of the CD28-superfamily costimulatory molecule. It was originally identified as a marker of T cell activation, and has since been found to have important roles in T cell proliferation and cytokine secretion [31, 32]. Anti-CTLA-4 can drive increased ICOS expression on T cells in clinical trials [33, 34], and ICOS upregulation on peripheral T cells is correlated with clinical responses to anti-CTLA-4 in bladder cancer [34]. We were interested in understanding if ICOS protein expression alone or in combination with OX-40 expression had prognostic significance in NSCLC. When we analyzed OX-40 expression in combination with ICOS-positive cell immune infiltrate, we did not find any significant improvement in survival, indicating that OX-40 is a stronger prognostic driver than ICOS positivity (Fig. 2d). This differential prognostic significance of OX-40 and ICOS expression could be explained by the fact that these two receptors belong to different classes of costimulatory molecules that have different roles in T cell activation. In fact, ICOS is a member of the CD28/CTLA-4 family; it is expressed on activated T cells and its ligand, B7H/B7RP-1, is expressed on B cells and in non-immune tissues after injection of lipopolysaccharide into animals [45, 46]. ICOS is important for T-cell dependent immune responses in vivo, as it is critical for efficient T cell priming and for the production of Th2 effector cytokines, in particular IL-4. Therefore, ICOS is a part of a mechanism by which immunity is directed towards humoral or inflammatory responses. OX-40 is a member of the TNFR-superfamily of receptors, which is not constitutively expressed on resting naïve T cells, unlike CD28. OX-40 is a secondary co-stimulatory immune checkpoint molecule, expressed 24 to 72 h following activation, that plays a crucial role in both Th1 and Th2 mediated reactions *in vivo*; its ligand, OX40L, is also not expressed on resting antigen presenting cells, but is expressed following their activation.

Among the top genes that showed significantly increased mRNA expression with high OX-40 protein expression, we observed increased gene expression of markers of T-cell inflammation and effector cell activation such as CD3, CD8, IFN-gamma, ICOS, CXCL9, CXCL10, CCL5, granzyme K [47]. Notably, ICOS, CCL5, CD3, CD8 are also included in published gene signatures associated with response to immunotherapeutic agents such as MAGE-A3 vaccination in NSCLC [48]. These findings suggest that OX-40 protein expression is a potential marker to select a subgroup of tumors that could be more responsive to immunotherapy strategies.

In conclusion, high OX-40 expression in the immune cell infiltrate is associated with better OS in patients with surgically resected stage I-III NSCLC. Furthermore, we observed that there is significant overlap in immune cells co-expressing OX-40 and other checkpoints such as PD-L1. Our study suggests the potential for OX-40 agonistic antibodies, currently in clinical development for NSCLC, to enhance the efficacy of existing checkpoint inhibition therapies.

## Supplementary information

**Additional file 1. Supplementary Data: Table S1.** Spearman’s correlation between OX-40 and other IHC immune markers. **Figure S1.** Overall survival Kaplan-Meier curves of OX-40 protein expression in squamous cell carcinoma histology (A) and adenocarcinoma histology (B) by median value. **Figure S2.** Overall survival Kaplan-Meier curves by OX-40 IHC level and CD3/CD8/OX-40 level (A), ICOS/OX-40 level (B), PD-L1/OX-40 level (C). (PDF 198 kb)

## Data Availability

The datasets used and/or analyzed during the current study are available from the corresponding author on reasonable request.
