# Supplementary material for: High OX-40 expression in the tumor immune infiltrate is a favorable prognostic factor of overall survival in non-small cell lung cancer
Source: J Immunother Cancer. 2019 Dec 16;7:351. doi: 10.1186/s40425-019-0827-2 (PMC6915970; doi:10.1186/s40425-019-0827-2)
Supplement: Supplementary file 1 — Additional file 1. Supplementary Data: Table S1. Spearman’s correlation between OX-40 and other IHC immune markers. Figure S1. Overall survival Kaplan-Meier curves of OX-40 protein expression in squamous cell carcinoma histology (A) and adenocarcinoma histology (B) by median value. Figure S2. Overall survival Kaplan-Meier curves by OX-40 IHC level and CD3/CD8/OX-40 level (A), ICOS/OX-40 level (B), PD-L1/OX-40 level (C). (PDF 198 kb) [file 40425_2019_827_MOESM1_ESM.pdf]

Table S1: Spearman's correlation between OX-40 and other IHC immune markers

	Rho	P-value
FOXP3	0.691	1.77e-15
CD3	0.620	6.34e-12
CD4	0.610	1.56e-11
PD1	0.572	4.97e-10
CD45RO	0.567	7.82e-10
CD8	0.565	9.17e-10
Granzyme B	0.558	1.62e-09
PD-L1	0.474	4.13e-06
CD68	0.429	8.37e-06
ICOS	0.150	0.157

Figure S1

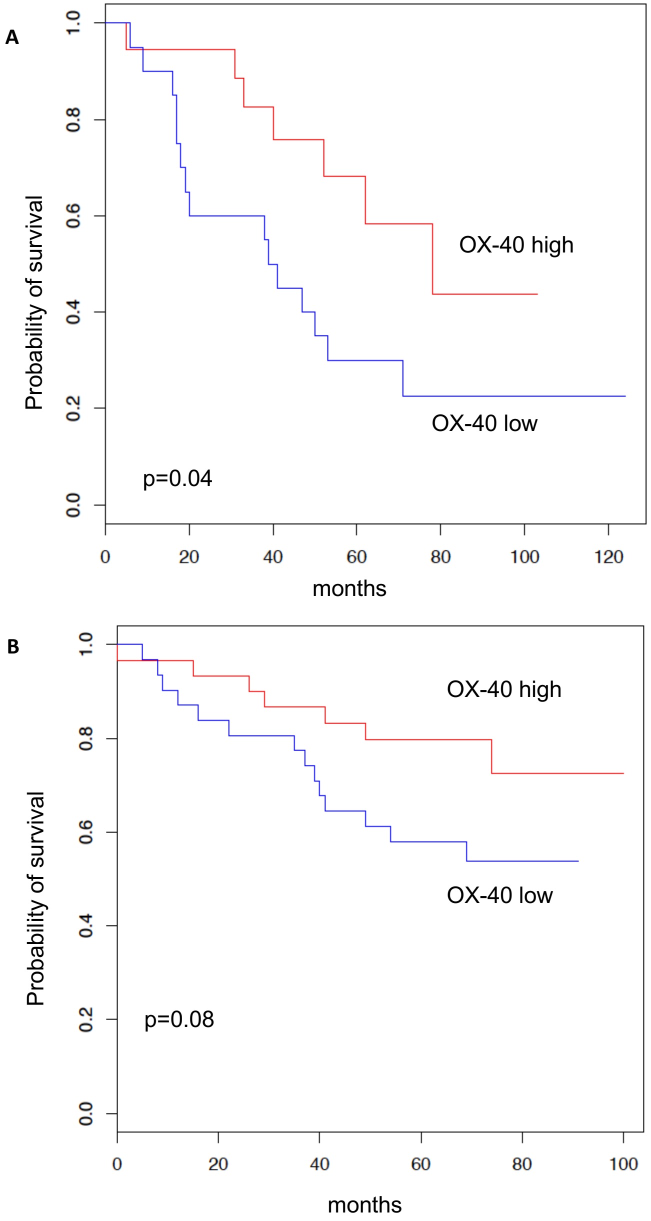

Figure S1. Overall survival Kaplan-Meier curves of OX-40 protein expression in squamous cell carcinoma histology (A) and adenocarcinoma histology (B) by median value.

Figure S2

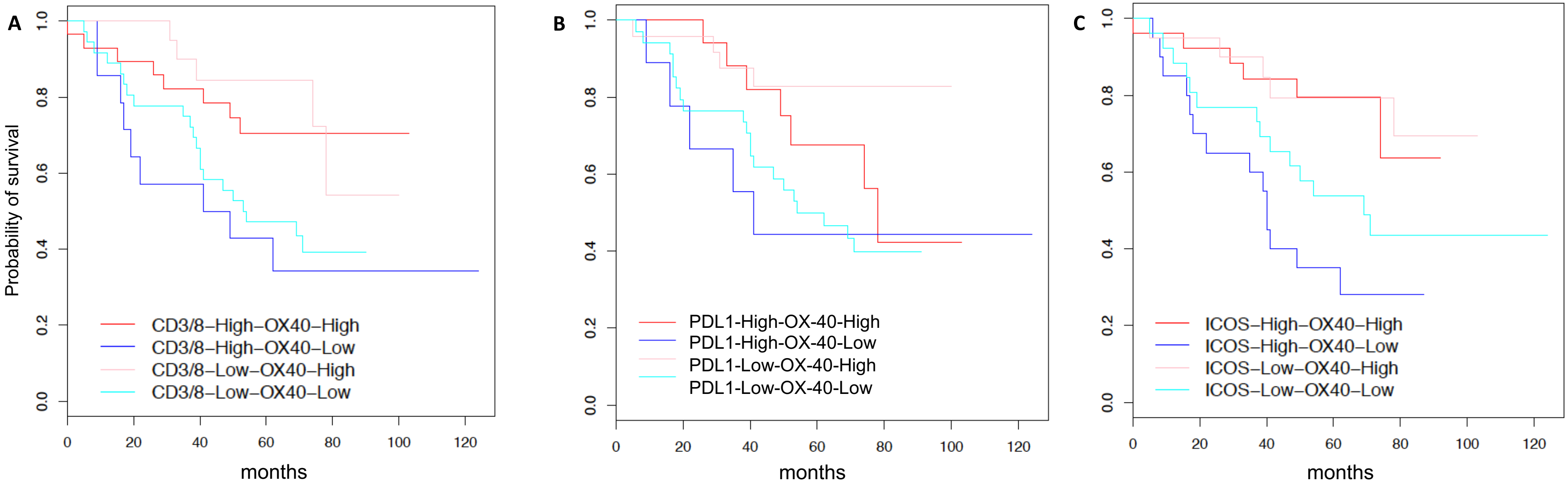

Figure S2. Overall survival Kaplan-Meier curves by OX-40 IHC level and CD3/CD8/OX-40 level (A), ICOS/OX-40 level (B), PD-L1/OX-40 level (C).
